# A 12-week double-blind randomized clinical trial of vitamin D_3_ supplementation on body fat mass in healthy overweight and obese women

**DOI:** 10.1186/1475-2891-11-78

**Published:** 2012-09-22

**Authors:** Amin Salehpour, Farhad Hosseinpanah, Farzad Shidfar, Mohammadreza Vafa, Maryam Razaghi, Sahar Dehghani, Anahita Hoshiarrad, Mahmoodreza Gohari

**Affiliations:** 1 Department of Nutrition, School of Public Health, Tehran University of Medical Sciences, Number 52, Alvand Street Arjantin Square, Tehran, Iran; 2 Obesity Research Center, Research Institute for Endocrine Sciences, Shahid Beheshti University of Medical Sciences, Floor 4th, Number 24, Parvaneh Street, Yemen Street, Chamran Exp, Tehran, Iran; 3 National Nutrition and Food Technology Research Institute, Shahid Beheshti University of Medical Sciences, Number 42, Arghavan Street, Farahzadi Boulevard, Shahrak-e Gharb, Iran; 4 Department of Biostatistics, Tehran University of Medical Sciences, Number 52, Alvand Street, Arjantin Square, Tehran, Iran

**Keywords:** Vitamin d3, Supplementation, Body fat mass, Obesity

## Abstract

**Background:**

Vitamin D concentrations are linked to body composition indices, particularly body fat mass. Relationships between hypovitaminosis D and obesity, described by both BMI and waist circumference, have been mentioned. We have investigated the effect of a 12-week vitamin D3 supplementation on anthropometric indices in healthy overweight and obese women.

**Methods:**

In a double-blind, randomized, placebo-controlled, parallel-group trial, seventy-seven participants (age 38±8.1 years, BMI 29.8±4.1 kg/m^2^) were randomly allocated into two groups: vitamin D (25 μg per day as cholecalciferol) and placebo (25 μg per day as lactose) for 12 weeks. Body weight, height, waist, hip, fat mass, 25(OH) D, iPTH, and dietary intakes were measured before and after the intervention.

**Results:**

Serum 25(OH)D significantly increased in the vitamin D group compared to the placebo group (38.2±32.7 nmol/L vs. 4.6±14.8 nmol/L; P<0.001) and serum iPTH concentrations were decreased by vitamin D3 supplementation (-0.26±0.57 pmol/L vs. 0.27±0.56 pmol/L; P<0.001). Supplementation with vitamin D3 caused a statistically significant decrease in body fat mass in the vitamin D group compared to the placebo group (-2.7±2.1 kg vs. -0.47±2.1 kg; P<0.001). However, body weight and waist circumference did not change significantly in both groups. A significant reverse correlation between changes in serum 25(OH) D concentrations and body fat mass was observed (r = -0.319, P = 0.005).

**Conclusion:**

Among healthy overweight and obese women, increasing 25(OH) D concentrations by vitamin D3 supplementation led to body fat mass reduction.

This trial is registered at clinicaltrials.gov as NCT01344161.

## Background

Obesity is a chronic condition of nutrients accumulation [1,2] in which excess energy aggregates in the form of fat mass [3]. Based on the thrifty genotype hypothesis [4], since metabolic efficiency is raised in negative energy states, important interactions between gene and obesogenic environment (including food abundance and low physical activity [5]) result in improper metabolic programming and epigenic change in utero; hence in this condition obesity is an inevitable outcome [6]. Fat mass distribution specifically visceral distribution, produces toxic milieu by initiating metabolic and inflammatory cascade, which is followed by endocrine, cardiovascular and malignant events. The risk of mortality rises synergistically with increase in BMI over than 30 kg/m2 [1].

Serum 25-hydroxyvitamin D concentrations are low in obese adults [7,8] and linked to components of body composition, particularly body fat mass [9,10]. Alterations in the vitamin D endocrine system have been reported in obesity [11,12]. Lumb et al believed that vitamin D is stored in adipose and muscle tissues after absorption, and is slowly released into the blood stream [13,14]. It was thought that vitamin D deficiency caused obesity, and is proposed that hypothalamus diagnoses low calcidiol concentrations in circulation and induces higher body set point by increase in appetite and decrease in energy consumption via stimulating Agouti Related Protein/Neuropeptide Y (AgRP/NPY) and suppressing pro-Opiomelanocortin/Cocaine- Amphetamine- Regulated Transcription (POMC/CART) pathway [15,16]. Wortsman et al confirmed insufficiency of vitamin D in the obese people indicating that they need to higher doses of vitamin D [17].

Evidence implies that dairy product consumption, and high calcium and/or vitamin D intakes can repress fatty acid synthase enzyme (FAS) by decreasing intracellular Ca + 2 in adiposities [18-21]. Recent literature reveals that vitamin D receptor (VDR) gene polymorphisms are associated with adiposity phenotypes [22]. It has been postulated that both 1,25(OH)2D and VDR have imperative roles in adipocyte differentiation [23,24]. The differentiation of pre-adipocytes to mature adipocytes *in vitro* is halted by 1,25(OH)2D3 [24]. Contrarily, high serum 1,25(OH)2 D concentrations may increase lipogenesis by stimulating of FAS [25].

Alterations in the vitamin D endocrine system are causally associated with augmented adiposity or result from augmented fat mass storage of vitamin D [17]. Accumulating evidence for involvement of vitamin D in fat mass metabolism [25] was the impetus for this clinical trial in which we tested the effect of vitamin D3 supplementation on body composition in overweight and obese women.

## Methods

### Subjects

We conducted the study between November 2009 and April 2010 in the Heart and Vascular Laboratory in Pharmacology Department of Tehran University of Medical Sciences, Tehran, Iran. Recruitment began in August 2009 by advertisements on university and ended in November 2009. The criteria for eligibility were age between 18-50 years old, a BMI ≥25 kg/m^2^, an apparently healthy status based on self-reports from the subjects, free from metabolic bone disease, gastrointestinal disease, diabetes mellitus, cardiovascular disease, renal disease, no medications, no vitamin supplements, none pregnant or lactating. We excluded individuals with changes in body weight more than 3 kg within last three months, following weight-loss programs, taking weight loss drugs, smoking and drinking alcohol. Of a total of 140 subjects initially selected, eighty five subjects who met the above inclusion criteria were recruited.

The present study was approved by the Ethics Committee of the Tehran University of Medical Sciences and Iranian Registry of Clinical Trial (registration no. IRCT138809092709N2) and written informed consent and subject assent were obtained.

### Design

Individuals were randomly allocated in a double-blind parallel manner from randomized number in an 85-person list; 42 women were assigned to the vitamin D group and 43 women to the placebo group. The vitamin D group had to take vitamin D3 supplement tablet of 25 μg/d as cholecalciferol; Merck Pharma GmbH, Germany, while the placebo group took tablet of 25 μg/d as lactose; Merck Pharma GmbH, Germany. The intervention was conducted for 90 days. To ensure and assess compliance, vitamin D supplements were issued at baseline, exchanged for a new package at both 4 wk and 8 wk, and returned to the research staff at post testing, and pills were counted later for compliance, which was 87.1% in the vitamin D and 87.4% in the placebo groups. To remain blinded, one research assistant who was not involved in data collection coordinated the supplement assignment schedule.

**Figure 1:**
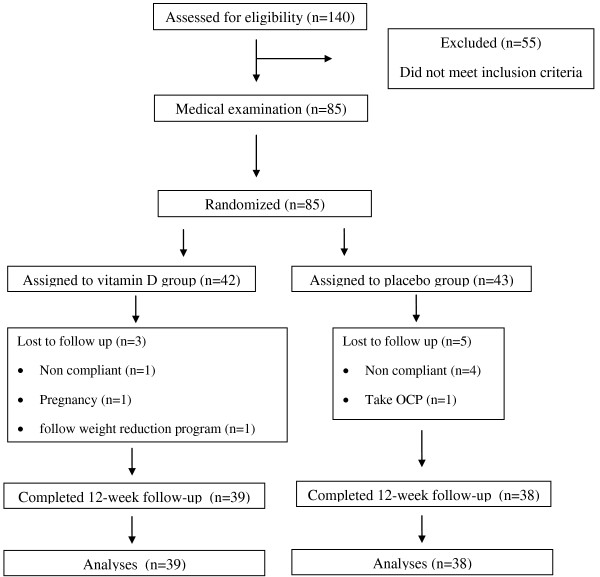
Follow of participants throughout the intervention.

In a per-protocol analysis, eight subjects were excluded during the intervention (Figure 1); in the placebo group, four subjects were unwilling to continue the 12-week intervention for personal reasons and another subject used oral contraceptive pills. In the vitamin D group, one subject followed a weight reduction program, one got pregnant and one was unwilling to continue the 12-week intervention for personal reasons. Eventually 77 subjects completed the study, 39 in the vitamin D group and 38 in the placebo group.

After a 12-h overnight fast, blood specimens were collected from the antecubital vein into the tubes. After centrifugation for 20 min (3000 g), the serum samples were frozen consecutively and stored at -80°C.

We assessed energy and nutrient intakes by 24 h food recall and validated food frequency questionnaires [26]. A nutritionist completed questionnaires during monthly face to face interviews. Because the Iranian food composition table (FCT) is incomplete (limited to only raw materials and few nutrients) [27], each food and beverage was analyzed for nutrient intake using Nutritionist IV software (Version 4.1, First Databank Division, The Hearst Corporation, San Bruno, CA) to assess macronutrient and micronutrient contents of foods. The Iranian FCT was used as an alternative for traditional Iranian food items, such as kashk, which are not included in the Food Composition Tables for USA (USDA FCT) [28]. The average of MET-minutes/week was calculated by multiplying the time of exercise by the respective metabolic equivalent task (MET) using the International Physical Activity Questionnaires (IPAQ) [29].

We measured body weight to the nearest 0.1 kg and height in light indoor clothes using a digital scale (model 763; Seca GmbH & Co, KG, Hamburg, Germany). Waist and hip circumference were measured on a horizontal plane at the level of the iliac crest by an Ergonomic Circumference Measuring Tape (model 201; Seca GmbH & Co, KG, Hamburg, Germany). Body mass index was calculated as weight in kilograms divided by the square of the height in meters. We assessed body fat mass and fat free mass by Bioelectrical Impedance Analysis (model 4000; Body Stat Quad Scan, Douglas Isle of Man, British Isles) after five minutes resting, with standard errors of estimate (accuracy) of 4.1%. All anthropometric indices were obtained using the WHO standard procedures [30].

### Biochemical measurements

Intact PTH was measured by immunoenzymo-metric assay (IEMA) (Immunodiagnostic Systems Ltd, Boldon, UK). Intra- and interassay CVs for intact PTH were 5.5%, and 8.3%, respectively. Serum 25(OH) D was measured by enzyme immunoassay (EIA) (Immunodiagnostic Systems Ltd, Boldon, UK). Intra- and interassay CVs for 25(OH) D were 6.9%, and 8.1%, respectively. Calcium was measured by colorimetric enzymatic (Pars Azmoon, Tehran, Iran), the kit expected range was 2.15-2.57 mmol/L. The assay sensitivity was 0.05 mmol/L, and intra- and interassay coefficients of variation were 2.4% and 3%, respectively. Phosphorus was measured by enzymatic photometric UV test (Pars Azmoon Co., Tehran, Iran); the kit expected range was 0.83-1.45 mmol/L. The assay sensitivity was 0.22 mmol/L, and intra- and interassay coefficients of variation were 3.2% and 4.1%, respectively.

### Statistical analysis

Descriptive statistics are presented as mean ± SD. We examined the normality of data by Kolmogorov-Smirnov and Shapiro-Wilk tests. All data had been normally distributed. For the primary analysis, we used an analysis of covariance (ANCOVA) to adjust mean differences on biochemical variables. Simple Pearson correlations were computed between changes in 25(OH) D and iPTH concentrations and body fat mass. All statistical analyses were performed using SPSS (version 16; SPSS Inc, Chicago, IL). All tests were two-sided and P values <0.05 were considered statistically significant.

## Results

**Figure 2:**
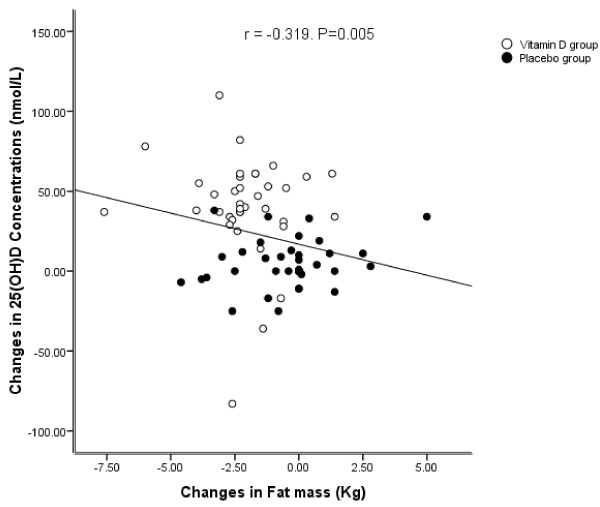
Relation between alterations of serum 25(OH) D concentrations and body fat mass.

**Figure 3:**
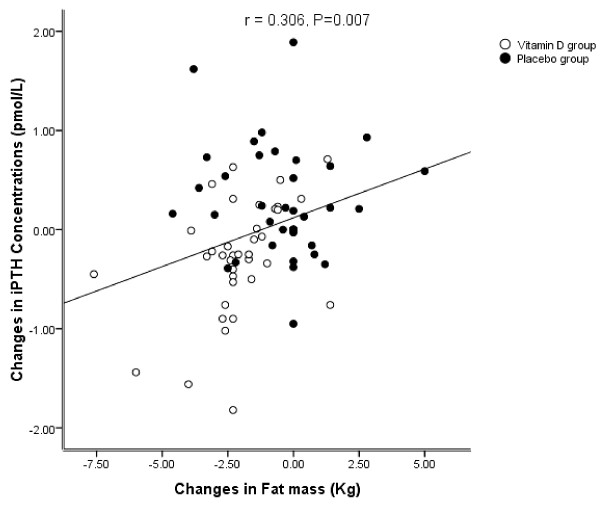
Relation between alterations of serum iPTH concentrations and body fat mass.

Participant characteristics are given in Table 1. Baseline characteristics were similar in the vitamin D and placebo groups (Table 1). Serum 25(OH) D concentrations increased to (75 ± 22 nmol/L vs. 51.5 ± 31 nmol/L; P<0.001), respectively in the vitamin D group, in comparison to the placebo group after 12 weeks (Table 2). Although serum iPTH concentrations decreased in the vitamin D group, these concentrations increased in the placebo group (-0.26 ± 0.5 pmol/L vs. 0.27 ± 0.5 pmol/L; P<0.001), respectively. In the vitamin D group, weight loss was (-0.3 ± 1.5 kg) whereas in the placebo group, it was (-0.1 ± 1.7 kg), differences were not statistically significant between the two groups. Waist circumference decreased in the vitamin D group, but increased in the placebo group (-0.3 ± 4.3 cm vs. 0.4 ± 4.1 cm), respectively. Hip circumference decreased (-0.39 ± 2.4 cm in the vitamin D group and -0.9 ± 2.4 cm in the placebo group), differences were not statistically significant between the two groups. Body fat mass decreased in the vitamin D and placebo groups (-2.7 ± 2 kg and -0.4 ± 2 kg; P<0.001). There were significant inverse correlations between changes in serum 25(OH) D concentrations and body fat mass (r = -0.319, P = 0.005) (Figure 2), and iPTH concentrations (r = -0.318, P = 0.005). A significant positive and correlation was observed between changes in serum iPTH concentrations and body fat mass (r = 0.32, P = 0.004) (Figure 3), while there were no significant correlations between serum 25(OH)D concentrations and body fat mass or iPTH concentrations.

**Table 1:** Baseline characteristics of subject groups who received vitamin D3 supplements (25 μ/d) or placebo before the intervention

**Characteristics**	**Vitamin D group**	**Placebo group**	**P-value**
Age (y)	38±7^2^	37±8	0.29
Body weight (kg)	73.9±10.2	75.1±11.9	0.61
Height (cm)	156.5±5.8	159.3±5.6	0.035
Waist circumference (cm)	89.9±8.7	91.2±12.1	0.59
Hip circumference (cm)	108±8.5	108.2±8.1	0.91
BMI (kg/m^2^)	30.1±3.9	29.5±4.4	0.54
Fat mass (kg)	30.2±6.9	29±8.7	0.53
Fat free mass (kg)	43.7±5.1	45.9±4.7	0.05
Physical activity (MET-minutes/week)	902±1245	702±996	0.43
Energy intake (kcal/d)	1866±927	2060±834	0.33
Carbohydrate intake (g/d)	280±134	329±140	0.12
Fiber intake (g/d)	16±9	18±10	0.23
Protein intake (g/d)	64±29	76±35	0.10
Fat intake (g/d)	55±44	49±24	0.43
Dietary calcium intake (mg/d)	873±586	677±386	0.08
Dietary vitamin D intake (μg/d)	0.53±0.6	0.39±0.37	0.22
25(OH) D (nmol/L)^2^	36.8±30	46.9±32	0.15
iPTH (pmol/L)^2^	1.4±0.7	1.4±0.7	0.84
Calcium (mmol/L)	2.2±0.09	2.3±0.1	0.004
Phosphorus (mmol/L)	1.1±0.1	1.1±0.1	0.6

^a^Mean ± SD (all such values).

^b^25(OH) D, 25-hydroxyvitamin D; PTH, parathyroid hormone. To convert 25(OH) D values to ng/mL, divide by 2.5. To convert PTH values to pg/mL, divide by 0.11.

**Table 2:** Anthropometric, dietary and serum variables in the subject groups after vitamin D3 supplementation and changes in variables between measurement periods

**Characteristics**	**Vitamin D group**		**Placebo group**		**P-value**^**a**^
	**Week 12**	**Change**^**b**^	**Week 12**	**Change**	
Body weight (kg)	73.5±10.4^3^	-0.3±1.5	75±12.3	-0.1±1.7	0.71
Waist circumference (cm)	89.5±8.8	-0.3±4.3	91.6±13	0.4±4.1	0.38
Hip circumference (cm)	107.6±7.9	-0.39±2.4	107.3±7.2	-0.9±2.4	0.36
BMI (kg/m^2^)	30±4	-0.13±0.6	29.5±4.6	-0.04±0.6	0.50
Fat mass(kg)	28.2±7.5	-2.7±2.1	28.6±8.9	-0.47±2.1	<0.001
Fat free mass (kg)	45.5±4.9	1.8±2.1	46.2±5	0.4±2.1	<0.001
Physical activity (METminutes/week)	892±1488	-10±1627	1081±1372	379±1137	0.23
Energy intake (kcal/d)	2010±1289	143.7±1358.4	1852±992	-208±920.9	0.32
Carbohydrate intake (g/d)	312±186	31.8±194.6	294±164	-34.3±143	0.23
Fiber intake (g/d)	16±12	1±11.7	14±7	-4.3±11.3	0.10
Protein intake (g/d)	72±53	7.8±54.3	66±32	-9.3±35.6	0.29
Fat intake (g/d)	53±43	-2.3±52.2	45±36	-4.2±39.3	0.48
Dietary calcium intake (mg/d)	829±533	-43.9±674.4	625±454	-51.8±509.5	0.18
Dietary vitamin D intake (μg/d)	0.4±0.47	-0.09±0.77	0.37±0.35	-0.04±0.52	0.70
25(OH) D (nmol/L)	75±22	38.2±32	51.5±31	4.6±14	<0.001
PTH (pmol/L)	1.2±0.5	-0.2±0.5	1.7±0.8	0.2±0.5	<0.001
Calcium (mmol/L)	2.2±0.1	-0.02±0.1	2.3±0.09	-0.02±0.1	0.81
Phosphorus (mmol/L)	0.9±0.09	-0.12±0.1	1±0.09	-0.09±0.1	0.21

^a^An analysis of covariance (ANCOVA) was used to adjust mean differences on all dependent variables.

^b^After 12 weeks.

Mean ± SD (all such values).

## Discussion

The present study shows that a 12 week supplementation with 25 μg vitamin D3 in overweight and obese women with mean serum 25(OH)D concentrations of 41.8±31.4 nmol/L decreases body fat mass, but does not affect body weight and waist circumference.

There is a large body of growing evidence showing that dairy products, and calcium and vitamin D intake play a role in the regulation of body fat mass [31-33]. Data also indicates that vitamin D may increase lean body mass [34] and inhibit the development of adipocytes. These effects of vitamin D may be mediated by 1,25(OH)2D3 or via suppression of PTH [35].

However, there are few clinical trials of vitamin D supplementation on body composition and most of them have assessed effects of combined calcium and vitamin D supplementation. Moreover, these studies are heterogeneous with regard to doses and types of vitamin D, lengths of follow up, outcome ascertainment methods, prevalence of vitamin D deficiency and other characteristics in studied subjects, which have led to inconsistent results. Our study is one of the few clinical trials which have investigated the effect of vitamin D3 supplementation in overweight or obese women with low 25(OH)D concentrations on body compositions. The 12 week vitamin D3 supplementation did not significantly affect body weight, waist or hip circumference. However, a modest fat mass reduction of 7% was associated with a significant increase of 25OHD levels by 103% and a significant decrease of PTH levels by 14%. The initial serum 25(OH)D concentrations were low in both groups.

Recently, in a double-blind, placebo-controlled trial in overweight and obese participants, Rosenblum et al [32] reported that after a 16 week calcium and vitamin D supplementation with either regular or reduced energy (lite) orange juice (three 240 mL glasses of orange juice fortified with 350 mg Ca and 100 IU vitamin D per serving), the average weight loss did not differ significantly between groups, but in the regular orange juice trial, the reduction of visceral adipose tissue (VAT) was significantly greater in the CaD group than in controls (−12.7 ± 25.0 cm^2^ vs. −1.3 ± 13.6 cm^2^; P = 0.024, respectively) and in the lite orange juice trial, the reduction of VAT was significantly greater in the CaD group than in controls (−13.1 ± 18.4 cm^2^ vs. − 6.4 ± 17.5 cm^2^; P = 0.039, respectively) after control for baseline VAT. They suggested that calcium and vitamin D supplementation contributes to a beneficial reduction of VAT. Dong et al [36] in a 16 week randomized, blinded, controlled clinical trial of 2000 IU vitamin D3 supplementation in forty nine black youth, evaluated the relation between 25(OH)D concentrations and total body fat mass by dual-energy x-ray absorptiometry. The experimental group compared with the controls reached significantly higher 25(OH)D concentrations at 16 wk (85.7±30.1 nmol/liter vs. 59.8±18.2 nmol/liter,P<0.001, respectively) and partial correlation analyses indicated that total body fat mass at baseline was significantly and inversely associated with 25(OH)D concentrations in response to the 2000 IU supplement (R = -0.46; P = 0.03). Zhou et al [37] in a large-scale, placebo controlled, double-blind, 4-year longitudinal clinical trial, investigated the effect of calcium and vitamin D supplementation on obesity in postmenopausal women, randomly assigned into one of three groups: 1) supplemental calcium (1400 mg/d or 1500 mg/d) plus vitamin D placebo (Ca-only group); 2) supplemental calcium (1400 mg/d or 1500 mg/d) plus supplemental vitamin D3 (1100 IU/d) (Ca + D group); or, 3) two placebos. No significant difference was observed for body mass index between groups, but changes in trunk fat (for Ca-only and Ca + D groups compared to the placebo group preserved lower trunk fat 2.4%, 1.4% vs. 5.4%, P = 0.015 at year 3) and trunk lean (for Ca-only and Ca + D groups preserved more trunk lean compared to the placebo group, -0.6%, -1.0% vs. -2.1%, P = 0.004 at year 4), were significantly different between groups. Major et al [38] conducted a randomized, double-blind, placebo-controlled study to compare the effects of a 15 week weight-reducing program (-700 kcal/d) coupled with a calcium plus vitamin D supplementation (600 mg elemental calcium and 5 mg vitamin D, twice a day or placebo), on the body fat of sixty-three overweight or obese women. The calcium + D supplementation did not induce statistically significant increase in fat mass loss. However, when analyses were limited to very low-calcium consumers only (initial calcium intake ≤600 mg/d), significant time × treatment interaction were observed in body weight (P<0·009), BMI (P<0·008) and fat mass (P<0.02). Waist circumference decreased in both groups and there were significant treatment effects (P = 0.03). Zittermann et al [39] investigated the effect of vitamin D (83 μg/d) on weight loss in overweight subjects with a mean 25(OH)D concentration of 30 nmol/L in a double-blind manner for 12 months while participating in a weight-reduction program. Their results showed that although weight loss was not affected significantly by vitamin D supplementation, waist circumference however decreased in both groups and there were significant treatment effects (P = 0.022). Body fat mass did not alter after the intervention.

It has been suggested that high levels of calcitrophic hormones such as 1α,25,dihydroxyvitamin D and iPTH can modulate intracellular Ca + 2 concentrations, so increasing Ca + 2 flux to adiposities, which stimulate fatty acid synthase enzyme, may increase lipogenesis and inhibit lipolysis [25]. According to this hypothesis, 1α, 25dihydroxyvitamin D is known as a key factor that provokes triglyceride accumulation in adiposities [18] a finding not confirmed by others [39]. Vitamin D insufficiency and secondary hyperparathyroidism lead to PTH related phospholipase C activation in adiposities, a process, which is followed by increase in intracellular calcium [40,41]. Chronic increase in [Ca + 2] may attenuate the ability of catecholamines in activating of lipolysis by increasing the activity of Ca + 2 related cAMP phosphodiesterase [42]. Meanwhile, with increasing of [Ca + 2], induction of fatty acid synthase is strengthened, which facilitates de novo lipogenesis [43]. Thus hyperparathyroidism can affect weight gain [44,45].

The primary limitation of our study is that we could not assess body composition by Dual X-Ray Absorptiometry (DXA) as a gold standard method. However, Bioelectrical Impedance Analysis is a validated and reliable method to assess body composition. A second limitation is that Resting Metabolic Rate (RMR) was not determined in subjects. Although our main goal was to examine the effect of vitamin D3 supplementation on body composition. A third potential limitation is not evaluating sun exposure, a confounding factor, which can not be completely ruled out. However, the subjects were requested not to use sunscreen during the intervention.

## Conclusions

To summarize, based on result of the 12 week vitamin D3 supplementation, we concluded body fat mass decreased in healthy overweight and obese women via increase in serum 25(OH) D and decrease in iPTH concentrations. Further investigation into whether vitamin D may play a role in regulation of body composition is warranted.

## Competing interests

None of the authors have any conflict of interest to declare.

## Authors’ contributions

As conceived of the original idea and aided with the experimental design, writing the final manuscript, and data interpretation. MR and SD carried out the all of the subject interviews, collection of data, data interpretation, and writing of the manuscript. FH, FS and MV carried out the study design, recruitment of patients, review of the original data and their compilation. AH and MG carried out data analysis, manuscript revision, critical revision of the manuscript for important scientific content. All authors read and approved the final manuscript.
